# Identification of Genes in *Candida glabrata* Conferring Altered Responses to Caspofungin, a Cell Wall Synthesis Inhibitor

**DOI:** 10.1534/g3.116.032490

**Published:** 2016-07-21

**Authors:** Anne G. Rosenwald, Gaurav Arora, Rocco Ferrandino, Erica L. Gerace, Maedeh Mohammednetej, Waseem Nosair, Shemona Rattila, Amanda Zirzow Subic, Ronda Rolfes

**Affiliations:** Department of Biology, Georgetown University, Washington, DC 20057

**Keywords:** caspofungin, echinocandins, cell wall integrity pathway, high affinity calcium uptake system

## Abstract

*Candida glabrata* is an important human fungal pathogen whose incidence continues to rise. Because many clinical isolates are resistant to azole drugs, the drugs of choice to treat such infections are members of the echinocandin family, although there are increasing reports of resistance to these drugs as well. In efforts to better understand the genetic changes that lead to altered responses to echinocandins, we screened a transposon-insertion library of mutants for strains to identify genes that are important for cellular responses to caspofungin, a member of this drug family. We identified 16 genes that, when disrupted, caused increased tolerance, and 48 genes that, when disrupted, caused increased sensitivity compared to the wild-type parental strain. Four of the genes identified as causing sensitivity are orthologs of *Saccharomyces cerevisiae* genes encoding proteins important for the cell wall integrity (CWI) pathway. In addition, several other genes are orthologs of the high affinity Ca^2+^ uptake system (HACS) complex genes. We analyzed disruption mutants representing all 64 genes under 33 different conditions, including the presence of cell wall disrupting agents and other drugs, a variety of salts, increased temperature, and altered pH. Further, we generated knockout mutants in different genes within the CWI pathway and the HACS complex, and found that they too exhibited phenotypes consistent with defects in cell wall construction. Our results indicate that small molecules that inhibit the CWI pathway, or that the HACS complex, may be an important means of increasing the efficacy of caspofungin.

*Candida glabrata* is a serious human pathogen; it is estimated that ∼20% of systemic candidiasis infections in the US are now caused by *C. glabrata*, making it the second most frequent cause after *C. albicans* (Richardson and Lass-Florl 2008). Immunocompromised patients, those receiving chemotherapy, immunosuppressive drugs, or infected with HIV/AIDS are particularly susceptible (Kale and Johnson 2005; Richardson and Lass-Florl 2008).

Treatment of *C. glabrata* infections is hampered by a dearth of effective drugs. Until recently, treatment of fungal infections was limited to amphotericin B and azoles, both of which target ergosterol—a key component of the fungal cellular membrane. However, known off-target effects, including organ toxicity, limit their effectiveness. Perhaps more problematic is the growing number of *C. glabrata* clinical isolates that are resistant to azoles, which has made this family of drugs ineffective against this pathogen (Krcmery and Barnes 2002). As a result, a significant fraction of patients, upwards of 35–40%, suffering from systemic *C. glabrata* infections die annually (Krcmery and Barnes 2002; Pfaller and Diekema 2004).

The development of the echinocandin class of drugs has helped to fulfill the need for more efficacious and safer antifungal drugs. This class exerts its fungicidal effects by disrupting cell wall synthesis, an ideal target because no comparable structure is present in human cells. Specifically, echinocandins (*e.g.*, caspofungin, micafungin, *etc*.) impact fungal cell wall synthesis by inhibiting the β-glucan synthases encoded by *FKS1*, *FKS2*, and, to a lesser extent, *FKS3* (Sucher *et al.* 2009), and represent the newest and most promising treatment modality available. Nevertheless, there are increasing reports of clinical resistance, chiefly as a result of “hot-spot” mutations in *FKS1*, and especially *FKS2* (Arendrup *et al.* 2012, 2013; Duran-Valle *et al.* 2012; Katiyar *et al.* 2012; Singh-Babak *et al.* 2012; Borghi *et al.* 2014; Pham *et al.* 2014). Echinocandin resistance in some *Candida* species, especially *C. albicans* is correlated with chitin overexpression (Walker *et al.* 2008, 2013). Further, other work has shown that stress from anti-fungals causes genetic instability in *Candida* species and other fungal pathogens, leading to multi-drug resistance (Shor and Perlin 2015).

*C. glabrata*, despite the genus name, is more closely related to *Saccharomyces cerevisiae* than to *C. albicans* (Kaur *et al.* 2005; Roetzer *et al.* 2011). Much of what is known about the genetics underpinning synthesis and maintenance of the cell wall comes from numerous studies in *S. cerevisiae* (Lesage and Bussey 2006; Orlean 2012), but there is an increasing focus on this pathway in pathogenic fungi as well (Dichtl *et al.* 2016). The fungal cell wall forms the outermost boundary for maintaining cell shape and permeability to macromolecules. Mechanical strength is provided by large complex macromolecules including glucans, chitin, and mannoproteins. The cell wall is dynamic, changing at different stages in the yeast life cycle (growth and budding), and in response to external cues (induction of sporulation among others) (Lesage and Bussey 2006; Orlean 2012). In *C. glabrata*, the cell wall appears to be similar, although generally thicker [up to 200 nm in *C. glabrata* (de Groot *et al.* 2008) compared to 120 nm in *S. cerevisiae* (Klis *et al.* 2014)] with a higher mannose/glucose ratio (0.81 compared to 0.57; de Groot *et al.* 2008), and 50% more protein than in *S. cerevisiae* cell walls (de Groot *et al.* 2008). In addition, to support the pathogenic lifestyle of *C. glabrata*, one of the key evolutionary adaptations is the expansion of the number of genes encoding cell surface adhesin proteins, which mediate interactions between the yeast cells and the host (Roetzer *et al.* 2011).

In *S. cerevisiae*, defects in or damage to the cell wall results in osmotic instability in turn activating the cell wall integrity (CWI) pathway, which ultimately controls transcription of cell wall-related genes such as FKS2/GSC2 (Levin 2011) (see Figure 1). Sensor-transducer proteins such as Slg1/Wsc1, Wsc2, Wsc3, Mid2, and Mtl1 detect damage (Kock *et al.* 2015). These signal to Rom1/2 (Ozaki *et al.* 1996; Manning *et al.* 1997), and guanine nucleotide exchange factors (GEFs) for Rho1 (Madden and Snyder 1998). Rho1 then activates the protein kinase Pkc1 (Antonsson *et al.* 1994; Watanabe *et al.* 1994; de la Fuente and Portillo 2000; Valdivia and Schekman 2003), which initiates a kinase cascade, in turn activating the kinase Bck1 (a MAPKKK), which then activates Mkk1/2 (MAPKKs) finally activating the MAP kinase Slt2/Mpk1 (Reinoso-Martin *et al.* 2003; Levin 2011). Slt2 phosphorylates several transcription factors, including Rlm1 (Jung *et al.* 2002) and Swi4/6 [(Nasmyth and Dirick 1991; Siegmund and Nasmyth 1996), also called SBF], turning on the expression of genes encoding proteins responsible for synthesis of the cell wall (Figure 1) (Levin 2011). *S. cerevisiae* strains lacking components of the CWI pathway, in particular strains lacking PKC1, BCK1, and SLT2, display pronounced sensitivity to caspofungin, demonstrating that the CWI pathway is required for caspofungin tolerance (Reinoso-Martin *et al.* 2003; Markovich *et al.* 2004). The high affinity calcium uptake system (HACS; Muller *et al.* 2001), a complex of at least three proteins, Mid1, Cch1, and Ecm7 (Martin *et al.* 2011), also signals to some of the genes responsible for cell wall synthesis via the calcinuerin-regulated transcription factor, Crz1 (Levin 2011; Martin *et al.* 2011). Thus regulation of cell wall biosynthesis is multi-layered, and receives information from a number of different pathways. Similar pathways exist in many pathogenic fungi of interest, including *C. glabrata*, other *Candida* species, *Aspergillus*, *Cryptococcus*, and *Pneumocystis* (Dichtl *et al.* 2016).

**Figure 1:**
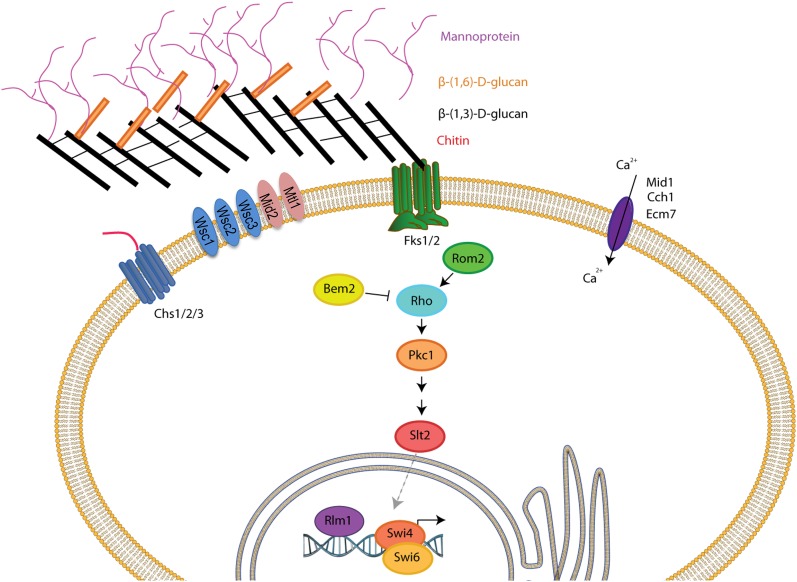
The cell wall, the cell integrity pathway, and the HACS complex (adapted from Levin 2011). Fks1 and Fks2 (green transmembrane proteins) are responsible for β-glucan synthesis {illustrated by black [β-(1,3)-d-glucan] and gold [β-(1,6)-d-glucan] bars} and are the targets for caspofungin action. The glucans form the inner cell wall. Mannoproteins are attached to the glucan layer and form the outer cell wall. Note that, in reality, the cell wall is closely apposed to the plasma membrane, containing the stress sensors, the three Wsc proteins, Mid2, and Mtl1. The chitin synthases, Chs1, Chs2, and Chs3 (dark blue), synthesize the chitin polymer that is found primarily at the bud neck between mother and daughter cells, and at bud scars on mother cells. Proteins of the cell wall integrity pathway, initiated by activation of the monomeric G protein Rho1, are illustrated in the center of the cartoon, resulting in activation of the transcription factors, Rlm1, and Swi4/Swi6. The plasma membrane complex for high affinity Ca^2+^ uptake, Cch1, Mid1, and Ecm7 is also illustrated here (dark purple).

In this study, we screened a collection of ∼ 27,000 *C. glabrata* strains created by insertion of a transposable element derived from Tn7, covering about 75% of the nonessential genes in the genome, for altered sensitivity to caspofungin (Castano *et al.* 2003). We identified 16 genes that, when disrupted, cause decreased susceptibility compared to wild type, and 48 genes that cause increased sensitivity. Isolated from this latter group were several *C. glabrata* genes that are putative orthologs of genes encoding proteins in the CWI pathway, and of the HACS complex of *S. cerevisiae*. When we made complete gene knockouts of the CWI and HACS genes isolated from the screen, as well as a number of other putative CWI orthologs, the strains exhibited cell wall defects, confirming that these genes encode proteins important for cell wall biosynthesis in *C. glabrata*.

## Materials and Methods

### Materials

Media reagents and chemicals were purchased from Thermo Fisher Scientific (Pittsburgh, PA) unless otherwise noted.

### Yeast strains and media

The collection of 27,000 *C. glabrata* Tn*7 URA3 hph* Km^R^ R6Kγ (Tn*7* UKR-H) mutagenized strains used in the screen were obtained from Brendan Cormack (Castano *et al.* 2003). The parental strain for this collection is BG14 (*ura3*Δ*(-85 + 932)*::*Tn903NeoR*) derived from the wild-type strain BG2 (Cormack and Falkow 1999). Strain BG2 was used as the reference strain for optimizing growth, drug treatment, and zymolyase assays. Yeast were grown at 30° in YPD medium (1% yeast extract, 2% Bacto-peptone, and 2% glucose) unless otherwise stated (Adams *et al.* 1997). Synthetic dextrose (SD) medium lacking uracil was used for selection of yeast transformants (Adams *et al.* 1997). To make solid media, 1.5% or 2% (w/v) agar was added. Strains were stored in 15% glycerol at –80°.

### Identification of the Tn7 insertion site in C. glabrata genomic DNA

The location of the transposon in each strain of interest was determined by sequencing DNA directly flanking either side of the transposable element. Specifically, genomic DNA was extracted (Adolph 1996), resuspended in TE buffer (10 mM Tris-HCl, 1 mM EDTA, pH 8.0), digested with *Xba*I (Promega, Madison, WI), and ligated by combining 1 µg of cut genomic DNA with T4 DNA ligase (New England Biolabs).

*Escherichia coli* strain BW23473 (Δ*lac-169 robA1 creC510 hsdR514* Δ*uidA*::*pir endA recA1*; Yale *E.coli* Stock Center, New Haven, CT) was used to maintain the Tn7-containing plasmids, which have an R6Kγ origin of replication requiring expression of the *pir* gene (Metcalf *et al.* 1996). Electrocompetent BW23473 were electroporated using a Gene Pulser (Bio-Rad, Hercules, CA) with the ligation mixture, then incubated in SOC medium (Hanahan 1983) for 1 hr at 37°, then plated on LB agar (Bertani 1951) containing 50 µg/ml kanamycin. Plasmids were isolated using the QIAprep Spin Miniprep kit (Qiagen, Valencia, CA).

To identify the genomic DNA flanking the transposable element in mutant strains, plasmids were sequenced outward from the transposon using the following primers: Tn7 Left (5′-ATAATCCTTAAAAACTCCATTTCCACCCCTCCCAG-3′), and Tn7 Right (5′-GACTTTATTGTCATAGTTTAGATCTATTTTGTTCAG-3′) (Green *et al.* 2012). Sequencing was performed by Genewiz (South Plainfield, NJ). The sequences obtained were compared to data in the *Candida* Genome Database (http://candidagenome.org; Inglis *et al.* 2012).

As a control, the presence of the Tn7 insertion was verified by PCR of genomic DNA. Oligonucleotides complementary to genomic DNA immediately up and downstream of the identified insertion site were used for PCR; amplification across the Tn7 insertion generates a larger band than the wild-type gene. PCR was also performed using one oligonucleotide specific to genomic DNA upstream of the insertion, and one specific to *URA3* within the Tn7 element; only PCR of DNA containing the transposon results in a band. Strains with evidence of a wild-type gene were eliminated from further consideration.

### Growth assays

Cells were grown in YPD medium at 30° to OD_600_ 0.8, then diluted 25-fold into a final volume of 0.25 ml in YPD containing varying concentrations of caspofungin (Merck, Whitehouse Station, NJ) in a 96-well plate, and placed at 30° under medium shaking. The plates were read at OD_600_ every 15 min for up to 10 hr using a GloMax microtiter plate spectrophotometer (Promega). The maximal specific growth rate (κ_max_) of each culture was calculated by fitting an exponential regression over the experimental points. These points were selected to yield a correlation coefficient (*R*^2^) higher than 0.9 when possible (in cultures where cells did not grow well the *R*^2^ value was 0.7–0.8). The κ constant from the equation y = y_0_e^(κ.^*^t^*^)^ was the maximal specific growth rate.

### Zymolyase sensitivity assays

Zymolyase assays were performed as previous described (Ovalle *et al.* 1998). Cells were grown to exponential phase in YPD medium, and then washed three times in 1× TE, and resuspended to an OD_600_ of 0.9 in 1× TE. Triplicate cultures were pipetted in to 96-well plates along with either 10% volume 1× TE (no-enzyme control), or with 10% volume 1× TE containing 100T zymolyase (Zymo Research, Irvine, CA) to a final concentration of 50 μg/ml. The OD_600_ was measured every 4 min for a total 160 min at room temperature using a GloMax instrument (Promega). Triplicate sample readings were averaged. Each experiment was completed at least three times.

### Gene ontology term analysis

Gene ontology (GO) term enrichment analysis was done with the BiNGO plug-in and the GOSlim terms for Cytoscape (Shannon *et al.* 2003; Maere *et al.* 2005). The *C. glabrata* gene identified was assumed to be the ortholog of the most closely related *S. cerevisiae* gene as identified by BLASTP (Altschul *et al.* 1990). GO terms for *S. cerevisiae* genes were downloaded from the GO consortium (http://www.geneontology.org/GO.downloads.annotations.shtml). The hypergeometric test was applied, and derived *P*-values were adjusted for multiple hypothesis testing by the method of Benjamini and Hochberg (1995). GO terms with adjusted *P*-values < 0.05 were considered significantly enriched. In addition, the gene list was submitted for analysis by FunSpec with and without Bonferroni correction (Robinson *et al.* 2002).

### Phenotype analysis

Mutants from the Tn7 collection selected for further analysis were stored at –80° in 15% glycerol in 96-well submasters. The mutants, either from the Tn7 collection, or the knockout mutants generated as described below, were diluted via a 96-pin replicator tool to new 96-well microtiter plates containing 200 µl of deionized water per well. Diluted cells were then replica printed onto plates containing solid YPD with and without one of the following: caspofungin, rapamycin (Sigma-Aldrich), Calcofluor white, tunicamycin (Sigma-Aldrich), caffeine (Sigma-Aldrich), Congo red (Thermo Fisher Scientific), or FK-506 monohydrate (Sigma-Aldrich). Specific concentrations used in a given experiment are listed in the corresponding figure legends.

In some experiments, diluted mutant strains were also replica printed onto YPD plates containing different salts including NaCl, KCl, LiCl, or CaCl_2_, with or without the addition of sorbitol. Effects of lower pH were also tested by preparing YPD plates buffered at pH 3.0 and 4.0 by the addition of phosphate-citrate buffers at pH 2.5 and 3.5, respectively, to YPD prior to autoclaving (Adams *et al.* 1997). Plates were incubated at 25° for 48 hr, except for plates incubated at 30°, 37°, or 42° to examine temperature sensitivity.

Growth of individual strains was evaluated semiquantitatively by 10-fold serial dilution followed by spotting onto solid medium. No growth even at the highest cell concentration was given a score of 0. Abundant growth even at the highest dilution was given a score of 4. All experiments were performed at least twice, and all data were evaluated by two of the authors.

Based on growth scores, hierarchal clustering analysis was performed using the hclust function of the stats package in the R programming environment, which utilizes the complete-linkage hierarchal clustering method (R Development Core Team 2013). In this agglomerative method, a distance matrix is calculated for the set of elements and each element is then placed in a cluster of its own, and clusters are then sequentially combined into larger clusters, until all elements end up in one cluster. At each step, the two clusters separated by the shortest distance are combined (Hartigan 1975). Heat maps showing expression fold change were generated using the gplots package developed for the R programming environment.

### Creation of C. glabrata knockout strains

A PCR-based method was used to create a gene knockout cassette to replace a gene with *URA3* in the BG14 background (Kuwayama *et al.* 2002). In each case, a piece containing the *URA3* gene flanked by 20 bp upstream and downstream sequences immediately proximal and distal to the open-reading frame to be replaced was stitched to an ∼500 bp upstream fragment and a ∼500 bp downstream fragment of the target gene to create a linear fragment large enough for homologous recombination to occur at the correct locus. The PCR reactions were performed with PrimeSTAR HS (Takara, Clontech, Mountain View, CA) following the manufacturer’s protocol.

First, amplification of the upstream, downstream, and *URA3* fragments was performed using genomic DNA with the appropriate primers designed from sequences in the *Candida* Genome Database (http://candidagenome.org; Inglis *et al.* 2012). Each PCR product was then size-fractionated on a 0.8% agarose gel in Tris-borate EDTA buffer (89 mM Tris-borate, pH 8.3, 2 mM EDTA). The corresponding band was excised and purified with GFX MicroSpin columns (GE Healthcare, Fairfield, CT). Second, a fusion PCR reaction was performed with PrimeSTAR HS (Takara) using purified upstream, downstream, and *URA3* fragments with primers complementary to the 5′ ends of the upstream and downstream fragments.

After confirming the size of the final fusion fragment, 50–100 ng of each fragment was transformed into *C. glabrata* strain BG14 (Ito *et al.* 1983). Transformants were selected on SD medium lacking uracil (Adams *et al.* 1997). Correct insertion of the fragment was verified by isolation of genomic DNA, followed by diagnostic PCR using an anti-sense oligonucleotide specific to *URA3* (5′-ATGTCTGCCCATTCTGCTATT-3′), and a sense oligonucleotide upstream of the outermost 5′ primer was used to construct the upstream fragment.

### Cloning of a C. glabrata homolog

In order to clone the *MID1* (CAGL0M03597g) gene into a CEN plasmid, the gene sequence with predicted 5′ and 3′ UTR regions was obtained from *Candida* Genome Database (http://candidagenome.org; Inglis *et al.* 2012). Primers were designed to amplify the entire gene sequence upstream and downstream of the predicted 5′ and 3′ UTR, and to add a *Not*1 restriction site to both ends of the resulting PCR product. Genes were amplified using 200 ng of BG14 genomic DNA, and 0.1 μM of each primer with Q5 DNA Polymerase (NEB). The resulting PCR product was purified using the MiniElute Reaction Cleanup Kit (Qiagen), and then digested with *Not*1 HF restriction enzyme (NEB). pRS410, an *ARS CEN* plasmid vector (Christianson *et al.* 1992) (obtained from Addgene, Cambridge, MA), was modified to replace the KanMX resistance cassette with NatMX. This was done by digesting the pCR2.1-NatMX vector with *Pme*1 and *Bgl*II (NEB) to excise the NatMX cassette, which was subsequently ligated to the pRS410 vector digested with *Bgl*II and *Nru*I (NEB). After successful ligation creating the pRS410-NatMX plasmid, this vector was then cut with *NotI* HF, and treated with calf intestinal alkaline phosphatase (NEB). Ligations were performed using T4 DNA ligase, and subsequently electroporated into electrocompetent DH10B cells. Candidate colonies were selected on LB plates containing 100 μg/ml ampicillin (Sigma-Aldrich) and 40 μg/ml X-gal (Sigma-Aldrich).

The plasmid as constructed above containing the *MID1* (CAGL0M03597g) gene was transformed into the *mid1*::*URA3* strain (Ito *et al.* 1983), and positive transformants were selected on YPD with 100 μg/ml nourseothricin/clonNat (Werner BioAgents, Jena, Germany). The empty pRS410-NatMX vector was used as a control.

### Data availability

The authors state that all data necessary for confirming the conclusions presented in the article are represented fully within the article.

## Results

### Identification of genes that, when disrupted, cause altered sensitivity to caspofungin

We screened a collection of 27,000 Tn7 insertion mutants for their responses to caspofungin, a member of the echinocandin family of anti-fungal drugs. We empirically found that two concentrations, 100 ng/ml and 200 ng/ml, in rich medium (YPD) allowed for selection of mutants both more sensitive and more tolerant than wild type (Supplemental Material, Figure S1). The entire collection, contained in 266 96-well plates, was replica-printed onto caspofungin-containing solid medium. The potential positives were screened a second time by streak-out on solid medium containing the same concentrations of caspofungin. From the set of ∼ 250 mutants that passed these initial tests, we prepared genomic DNA and created plasmids including the Tn7 replicon. The modified Tn7 element contains an origin of replication for *E. coli* as well as selectable markers for *E. coli* and yeast. Genomic DNA derived from a Tn7 insertion strain can thus be recovered as a plasmid and propagated in *E. coli*. Sequencing of the plasmid from regions within the Tn7 element into the surrounding genomic DNA identifies the region interrupted by the transposon (Castano *et al.* 2003). The resulting plasmids were sequenced, and strains where the transposon had inserted between two genes, or where it appeared there was an unusual recombination event, were eliminated, keeping only those strains where the Tn7 replicon had inserted into the middle of an open reading frame.

Many of the strains were found to have insertions in the same gene, often at different sites. Each of these strains had the same phenotype with respect to caspofungin (*i.e.*, either all sensitive or all tolerant). We were therefore confident that these genes represented authentic hits. However, many other genes were represented only by a single example. For these strains, we examined whether there was evidence for nonhomologous recombination by diagnostic PCR. If any strains appeared to have a wild-type copy of the gene of interest, in addition to the disrupted copy, they were eliminated from further analysis. At the end of this screening process, we identified a total of 64 genes that, when disrupted, resulted in a strong phenotype with respect to caspofungin, 48 that caused increased sensitivity, and 16 that caused tolerance (Table 1 and Table 2).

**Table 1:** Genes whose disruption confers tolerance to 200 ng/ml caspofungin in YPD medium

*C gla* Locus Tag	*S. cerevisiae* Homolog	Gene Name	Description of Gene Product (Engel *et al.* 2016)
Cell wall assembly			
CAGL0E02629g	YOR002W	ALG6	α-1,3 Glucosyltransferase
CAGL0L00693g	YIL049w	DFG10	Polyprenol reductase
CAGL0G00286g	YMR307W	GAS1	GPI-anchored β-1,3 glucanosyltransferase
Cytoskeleton and vesicular transport			
CAGL0L11814g	YER166w	DNF1	Aminophospholipid translocase (flippase)
CAGL0M08052g	YEL022w	GEA2	Arf GEF
CAGL0A02629g	YHR108w	GGA2	Regulates Arf1 and Arf2 to facilitate Golgi trafficking
CAGL0B04631g	YOR109W	INP53	Polyphosphatidylinositol phosphatase
Cell signaling/response to stress			
CAGL0K04169g	YGR040w	KSS1	MAPK involved in filamentous growth and pheromone response
CAGL0J01870g	YGL167C	PMR1	High affinity Ca^2+^/Mn2+ P-type ATPase/transport into Golgi
Transcription regulation/DNA and RNA repair and modification			
CAGL0F05379g	YDR206w	EBS1	Involved in translation inhibition and nonsense-mediated decay
CAGL0K11132g	YDR240c	SNU56	Component of U1 snRNP required for mRNA splicing via spliceosome
Miscellaneous and unknown functions			
CAGL0D05324g	YBR255W	MTC4	Unknown function; β-1,6 glucan excretion increased in null
CAGL0K04147g	YGR038w	ORM1	Unknown function/response to unfolded protein
CAGL0C02717g	YAL009W	SPO7	Putative regulatory subunit of Nem1p-Spo7p phosphatase holoenzyme
CAGL0C04587g	YJR098c	—	Unknown function, found in highly purified mitochondria
CAGL0K08008g	YPR089W	—	Unknown function, interacts genetically with ERG11 and physically with Hsp82

**Table 2:** Genes whose disruption confers sensitivity to 100 ng/ml caspofungin in YPD medium

*C gla* Locus Tag	*S. cerevisiae* Homolog	Gene Name	Description of Gene Product (Engel *et al.* 2016)
PKC/cell wall integrity pathway			
CAGL0I06512g	YER155c	BEM2	RhoGAP involved in the control of cytoskeleton organization
CAGL0C05599g	YDL240w	LRG1	Putative GAP appears to specifically regulate β-1,3 glucan synthesis
CAGL0J03828g	YPL140C	MKK1	MAPKK involved in control of cell integrity
CAGL0G09559g	YOR188w	MSB1	Unknown function; may be involved in positive regulation of β-1,3 glucan synthesis and the Pkc1-MAPK pathway
CAGL0G01320g	YNL053W	MSG5	Dual-specificity protein phosphatase/ regulates Slt2
CAGL0E04620g	YDR055w	PST1	Cell wall protein that contains a putative GPI-attachment site
CAGL0J00539g	YHR030c	SLT2	Serine/threonine MAP kinase; regulates cell wall integrity
CAGL0A04565g	YER111c	SWI4	DNA binding component of the SBF complex
Cytoskeleton and vesicular transport			
CAGL0J04312g	YBL017C	PEP1	Type I transmembrane sorting receptor for multiple vacuolar hydrolases
CAGL0E05302g	YOR329c	SCD5	Protein required for normal actin organization and endocytosis
CAGL0G05786g	YDL212w	SHR3	Endoplasmic reticulum packaging chaperone
CAGL0K05291g	YPR032W	SRO7	Effector of Rab GTP-binding protein Sec4
High affinity Ca^2+^ influx (HACS)			
CAGL0B02211g	YGR217W	CCH1	Voltage-gated high-affinity Ca^2+^ channel
CAGL0M00748g	YLR443W	ECM7	Role in Ca^2+^ uptake
CAGL0M03597g	YNL291C	MID1	Ca2+-permeable cation channel required for Ca^2+^ influx
Mannan synthesis			
CAGL0K11231g	YDR245w	MNN10	Subunit of a Golgi mannosyltransferase complex
CAGL0M02871g	YJL186w	MNN5	α-1,2-mannosyltransferase
CAGL0J08734g	YAL023c	PMT2	Protein O-mannosyltransferase
CAGL0B02321g	YML115C	VAN1	Component of the mannan polymerase I
Cell signaling/response to stress			
CAGL0L11110g	YLR433c	CNA1	Calcineurin A; regulates Crz1
CAGL0K01507g	YDL035c	GPR1	Plasma membrane GPCR; nutritional state sensor
Vacuole			
CAGL0F06347g	YMR054W	STV1	Subunit a of the vacuolar-ATPase V0 domain
Transcriptional/translational regulation			
CAGL0M06831g	YNL027w	CRZ1	TF, activates transcription of stress response genes
CAGL0J10120g	YNL068c	FKH2	Forkhead family transcription factor
CAGL0M13431g	YHR187w	IKI1	Subunit of hexameric RecA-like ATPase Elp456 elongator subcomplex
CAGL0C04477g	YDL005c	MED2	Subunit of the RNA polymerase II mediator complex
CAGL0M07029g	YCR077C	PAT1	Deadenylation-dependent mRNA-decapping factor
CAGL0J03322g	YER082C	UTP7	Nucleolar protein/processing of pre-18S rRNA
CAGL0H04367g	YML076C	WAR1	Homodimeric Zn2Cys6 zinc finger transcription factor
DNA replication/repair/cell cycle regulation			
CAGL0D06028g	YJR053w	BFA1	Component of the GTPase-activating Bfa1-Bub2 complex
CAGL0I02222g	YHR164C	DNA2	Tripartite DNA replication factor
CAGL0K09438g	YOR144c	ELG1	Subunit of a complex important for DNA replication/genome integrity
CAGL0A03300g	YGL192w	IME4	mRNA N6-adenosine methyltransferase required for entry into meiosis
CAGL0D05786g	YLL002w	RTT109	Histone acetyltransferase
CAGL0A03432g	YLR032w	RAD5	DNA helicase
CAGL0E02475g	YOL004W	SIN3	Component of the Sin3-Rpd3 histone deacetylase complex
Proteosome			
CAGL0G09493g	YOR191w	ULS1	Protein involved in proteolytic control of sumoylated substrates
Miscellaneous and unknown functions			
CAGL0E03355g	YLL015W	BPT1	ABC type transmembrane transporter of MRP/CFTR family
CAGL0J08910g	YLR213c	CRR1	Putative glycoside hydrolase of the spore wall envelope
CAGL0M05511g	YBR207w	FTH1	Putative high affinity iron transporter
CAGL0E03311g	YGR163w	GTR2	Putative GTP binding protein that negatively regulates Ran/Tc4 GTPase cycle
CAGL0I07447g	YOL103w	ITR2	Myo-inositol transporter
CAGL0J07436g	YNL231c	PDR16	PITP controlled by the multiple drug resistance regulator Pdr1
CAGL0H04213g	YML081W	TDA1	Protein kinase with unknown role; localizes to cytosol and nucleus
CAGL0J09966g	YNL064c	YDJ1	Type I HSP40 cochaperone
CAGL0M08250g	YKL175w	ZRT3	Vacuolar membrane zinc transporter
CAGL0L05060g	YKL075c	—	Unknown function; localizes to cytosol
CAGL0K03377g	YMR102C	—	Unknown function; paralog of Dgr2

Few genes in the *C. glabrata* genome have been annotated beyond pointing to a highly similar gene in the *S. cerevisiae* genome. The corresponding *S. cerevisiae* gene names were submitted for GO term analysis using the BinGO package (Shannon *et al.* 2003; Maere *et al.* 2005). Table S1A shows the biological processes in which the genes are involved: transport (including vesicle transport), signal transduction, response to chemical stimulus, and importantly, cell wall organization. When the gene names were submitted to the FunSpec ontology tool (Robinson *et al.* 2002), the resulting output also suggested many of the genes encode modulators of ion homeostasis in cells, especially Ca^2+^ homeostasis (Table S1B).

### Phenotypes of selected Tn7 mutants

**Figure 2:**
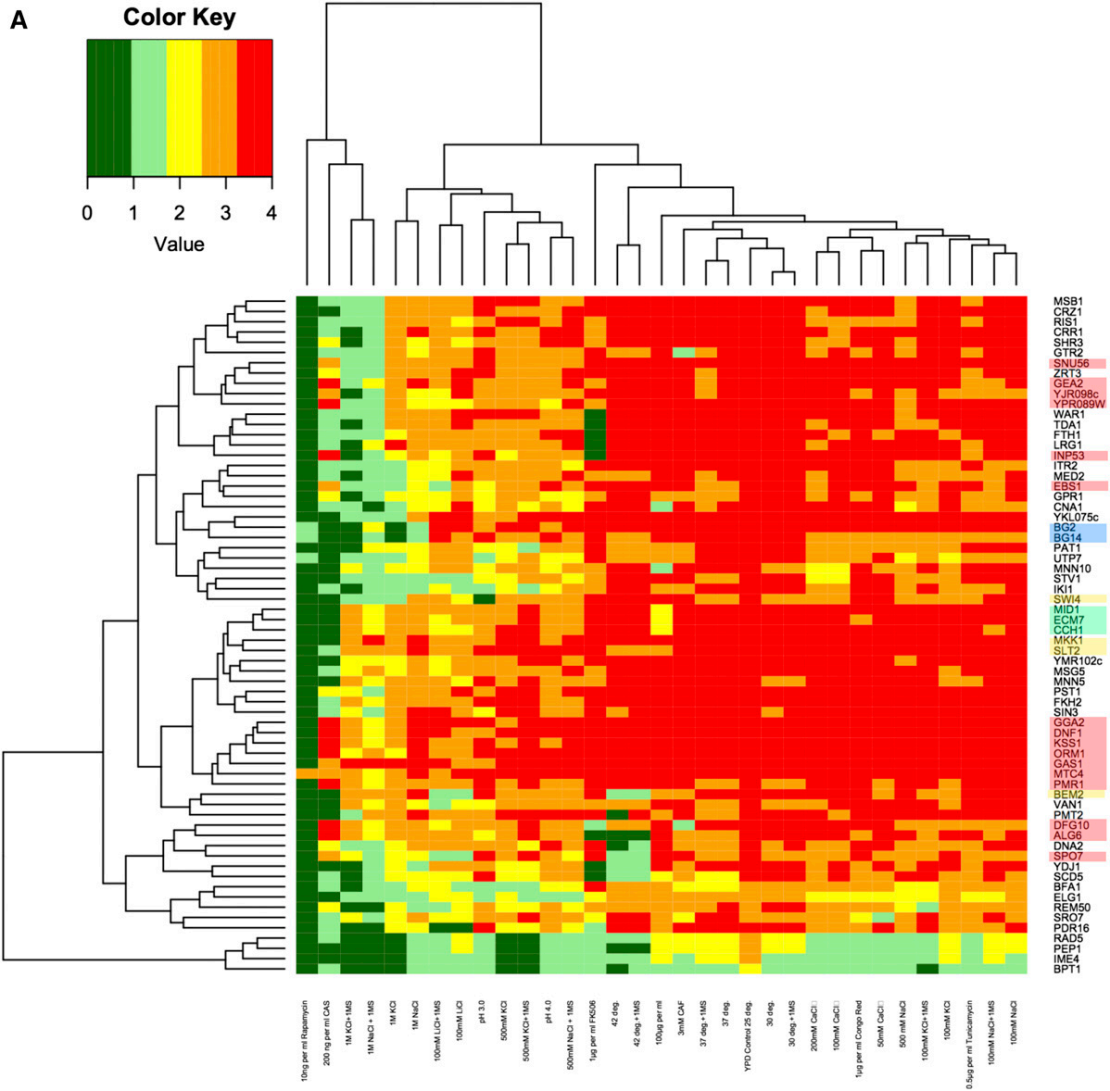

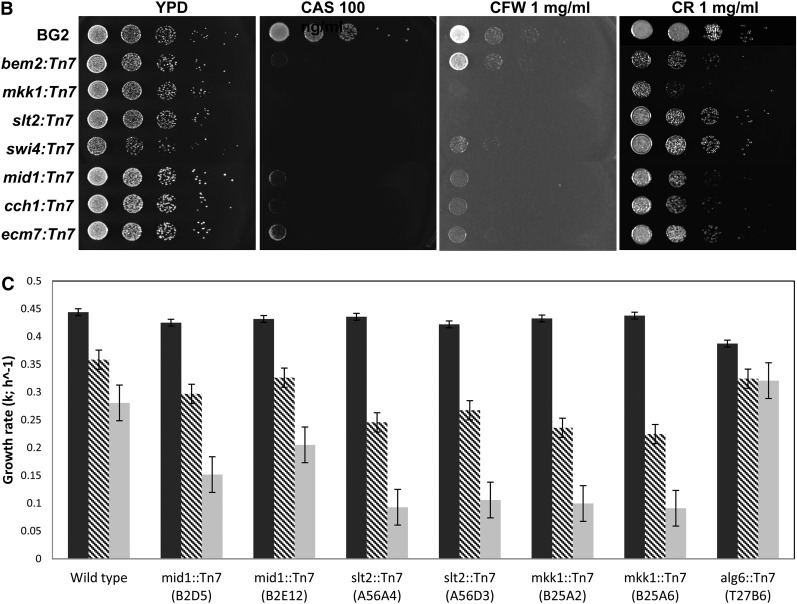
Phenotypes of the 64 Tn7 disruption mutants. (A) Sixty-four strains, each with one of the genes of interest disrupted by Tn7, were grown, diluted, and then replica-printed onto YPD medium, without or with the addition of a variety of drugs or salts as described in *Materials and Methods*. All plates were incubated for 2 d, then were photographed and scored. All plates were incubated at 25°, except for plates incubated at 30°, 37°, or 42°, with or without the addition of 1 M sorbitol. Additions included: 100 mM NaCl, with and without 1 M sorbitol, 500 mM NaCl, with and without 1 M sorbitol, 1 M NaCl, with and without 1 M sorbitol, 100 mM LiCl, with and without 1 M sorbitol, 100 mM KCl, with and without 1 M sorbitol, 500 mM KCl, with and without 1 M sorbitol, 1 M KCl, with and without 1 M sorbitol, 50 mM CaCl_2_, 100 mM CaCl_2_, 200 mM CaCl_2_, 1 μg/ml Congo red, 10 ng/ml rapamycin, 0.5 μg/ml tunicamycin, YPD at pH 3.0, YPD at pH 4.0, 100 μg/ml calcofluor white, 3 mM caffeine, 200 ng/ml caspofungin, or 1 μg/ml FK506. Strains were compared to the parental wild-type strains BG2 and BG14 (BG2 *ura3*). The raw data (in Table S3) were summarized as a heat map. Strains that grew well under a given condition were scored as 4+ and assigned the color red as shown on the schematic; strains that grew poorly were scored as 0 and assigned the color green. The names of disrupted *C. glabrata* orthologs of *S. cerevisiae* genes in each strain are shown at the left; the growth conditions that cluster together are shown at the top. The two wild-type strains are highlighted in blue, the genes that, when disrupted, cause caspofungin resistance are highlighted in red (all others are caspofungin sensitive). Members of the HACS complex are highlighted in green, and members of the CWI pathway are highlighted in yellow. Phenotypes of each strain with respect to the different growth conditions were verified at least twice. This figure represents one set of growth experiments. (B) Growth of mutants on solid medium with and without cell wall disrupting agents. Tn7 disruption mutants for putative *C. glabrata* CWI pathway and HACS complex genes, *bem2:Tn7 (A96B09)*, *mkk1:Tn7 (B25A2)*, *slt2:Tn7 (A56A4)*, *swi4*Δ*:Tn7(A4F9)*, *mid1:Tn7 (B2D5)*, *cch1:Tn7 (T77F2)*, and *ecm7:Tn7 (T66C3)* were spotted as 10-fold dilutions on YPD plates containing 100 ng/mL caspofungin (CAS), 1 mg/ml calcofluor white (CFW), 1 mg/ml Congo red (CR). The parental strain BG2 served as a comparison for growth. (C) Effects of caspofungin on growth of selected Tn7 disruption mutants in liquid culture. Log-phase cells were diluted as described in *Materials and Methods* in YPD alone (black bars) or with caspofungin [4 ng/ml (hatched bars) or 8 ng/ml (gray bars)]. OD_600_ was measured at 15-min intervals for several hours to measure growth. Growth rate, κ was calculated as described. *Cg MID1* encodes a putative member of the HACS complex, while *Cg MKK1* and *Cg SLT2* encode putative kinases of the CWI pathway. *Cg ALG6* encodes a putative α-1,3 glucosyltransferase. Disruptions of *Cg MID1*, *Cg SLT2*, and *Cg MKK1* all result in sensitivity to caspofungin, while disruption of *Cg ALG6* is unaffected by the presence of the drug.

Tn7-disruption mutants in each of the 64 genes identified in the screen were further characterized. The phenotypes tested by replica-printing onto solid medium included sensitivity to a variety of cell wall disrupting agents in addition to caspofungin, including Congo red and Calcofluor white; a number of other drugs including tunicamycin (a glycosylation inhibitor), rapamycin and caffeine (TOR inhibitors), and FK506 (a calcineurin inhibitor); salts including KCl, NaCl, LiCl, and CaCl_2_; different pH treatments; and different temperatures (summarized in Figure 2A); for complete data set, see Table S3.

Most of the mutant strains, like the wild-type parental strains, were resistant to both cell wall perturbing dyes, Calcofluor white and Congo red (Figure 2A and Table S3). A few mutant strains exhibited sensitivity to caffeine, while nearly all mutants exhibited sensitivity to rapamycin, except for the strain with an insertion in *Cg MTC4* (CAGL0D05324g). Most mutants were sensitive to tunicamycin, and, finally, the majority of mutants were resistant to the concentration of FK506 tested (Table S3).

Since FunSpec analysis revealed that genes involved in ion homeostasis and calcium transport were represented in our gene set, we explored the ion tolerance of mutants by growth on media containing NaCl, KCl, LiCl, and CaCl_2_. Sorbitol was added as an osmotic stabilizer to examine whether growth of mutants exhibiting sensitive phenotypes could be rescued; however, in most cases, sorbitol exacerbated sensitivity. Most of the mutants exhibited unimpeded growth in the presence of NaCl and KCl at levels of 100 mM and 500 mM. However, in the presence of 1 M NaCl or KCl, a greater diversity in growth effects was observed. A few mutants exhibited moderate to high sensitivity to most ionic conditions while another group of mutants showed a high degree of resistance to most conditions except the presence of caspofungin (Table S3), including the orthologs of the HACS complex genes and the orthologs of some genes encoding members of the CWI pathway.

The results of the 33 different phenotype tests were subjected to clustering analysis (Figure 2A), which revealed several broad groupings. First, the wild type strains (BG2 and its *ura3* derivative, BG14 the parent of the Tn7 collection, highlighted in blue on the phylogram) clustered together, demonstrating the robustness of this approach. The smallest group contained mutant strains that were sensitive to most conditions, except for growth at 25° in rich medium (at the bottom of Figure 2A); these four mutants generally grew poorly under most stresses, and were not examined further. The caspofungin-resistant mutants clustered together for the most part (highlighted in red, Figure 2A) while caspofungin-sensitive mutants were dispersed among the clusters.

### Disruption of CWI pathway and HACS genes: further characterization of the caspofungin phenotype

Mutant strains with disruptions of genes encoding orthologs of kinases in the cell wall integrity (CWI) pathway (Figure 1), *Cg SLT2* (CAGL0J00539g) and *Cg MKK1* (CAGL0H05621g), both sensitive to caspofungin, clustered in our analysis (highlighted in yellow, Figure 2A). In addition, we identified two additional genes homologous to *S. cerevisiae* CWI pathway orthologs, *Cg BEM2* (CAGL0I06512g; encoding RhoGAP) and *Cg SWI4* (CAGL0A04565g ; a transcription factor) (also highlighted in yellow, Figure 2A). Finally, we observed that strains with disruptions in one of the three genes encoding orthologs of voltage-gated high-affinity Ca^2+^ channel genes grouped together (Martin *et al.* 2011) [*Cg CCH1* (CAGL0B02211g), *Cg ECM7* (CAGL0M00748g), and *Cg MID1* (CAGL0M03597g); highlighted in green, Figure 2A].

We next examined the presumptive CWI [*Cg bem2:Tn7* (CAGL0I06512g), *Cg mkk1:Tn7* (CAGL0J03828g), *Cg slt2:Tn7* (CAGL0J00539g), and *Cg swi4:Tn7* (CAGL0A04565g)] and HACS [*Cg mid1:Tn7* (CAGL0M03597g), *Cg cch1:Tn7* (CAGL0B02211g), and *Cg ecm7:Tn7* (CAGL0M00748g)] mutant strains in more detail first, by growth on solid medium (Figure 2B) and then by growth in liquid culture (Figure 2C). On solid medium, all of the Tn7 insertion mutants tested grew reasonably well in the absence of drugs [although the *Cg swi4:Tn7* (CAGL0A04565g) strain was noticeably slower]. All the mutants were sensitive to caspofungin and Calcofluor white, although on Calcofluor white, it was possible to distinguish a greater variety of phenotypes. Finally, several of these strains were quite sensitive to Congo red, including the *Cg bem2:Tn7* (CAGL0I06512g) and *Cg mkk1:Tn7* (CAGL0J03828g) strains. In contrast, the *Cg slt2:Tn7* (CAGL0J00539g) and *Cg swi4:Tn7* (CAGL0A04565g) strains grew as well as the wild-type strain in the presence of Congo red; the remaining strains had intermediate phenotypes (Figure 2B).

We then examined the effect of caspofungin on growth of two independent *Cg SLT2* (CAGL0J00539g) and *Cg MKK1* (CAGL0J03828g) mutants in liquid medium. As can be seen in Figure 2C, while wild type was little affected by the lower concentration of caspofungin, the growth of all four mutant strains was adversely affected. We also examined two independent strains with insertions into *Cg MID1* (CAGL0M03597g), encoding a subunit of the HACS complex, and found these were also more sensitive to caspofungin than wild type. In contrast, a strain with an insertion in *Cg ALG6* (CAGL0E02629g), putatively encoding α-1,6 glucosyltransferase, obtained from the screen as a caspofungin-resistant mutant, was unaffected by the presence of caspofungin in this assay.

### Phenotypes of C. glabrata deletion mutants

To further explore the CWI pathway in *C. glabrata*, we constructed deletions of each of the four putative CWI genes obtained in the screen [*i.e.*, *Cg BEM2* (CAGL0I06512g), *Cg MKK1* (CAGL0J03828g), *Cg SLT2* (CAGL0J00539g), and *Cg SWI4* (CAGL0A04565g)], and four other presumptive CWI pathway genes, *Cg BCK1* (CAGL0L03520g , a third Ser/Thr kinase), *Cg RLM1* (CAGL0H05621g , a transcription factor), *Cg ROM2* (CAGL0G04873g , Rho GEF), and *Cg SLG1* (CAGL0F01507g , a plasma membrane sensor) (Levin 2011). These genes represent all stages of the pathway, from cell-surface sensing to regulation of transcription (Figure 1). We also constructed deletions of *Cg MID1* (CAGL0M03597g), *Cg CCH1* (CAGL0B02211g), and *Cg ECM7* (CAGL0M00748g)—all members of HACS complex (for the complete set of deletion strains constructed see Table S2).

**Figure 3:**
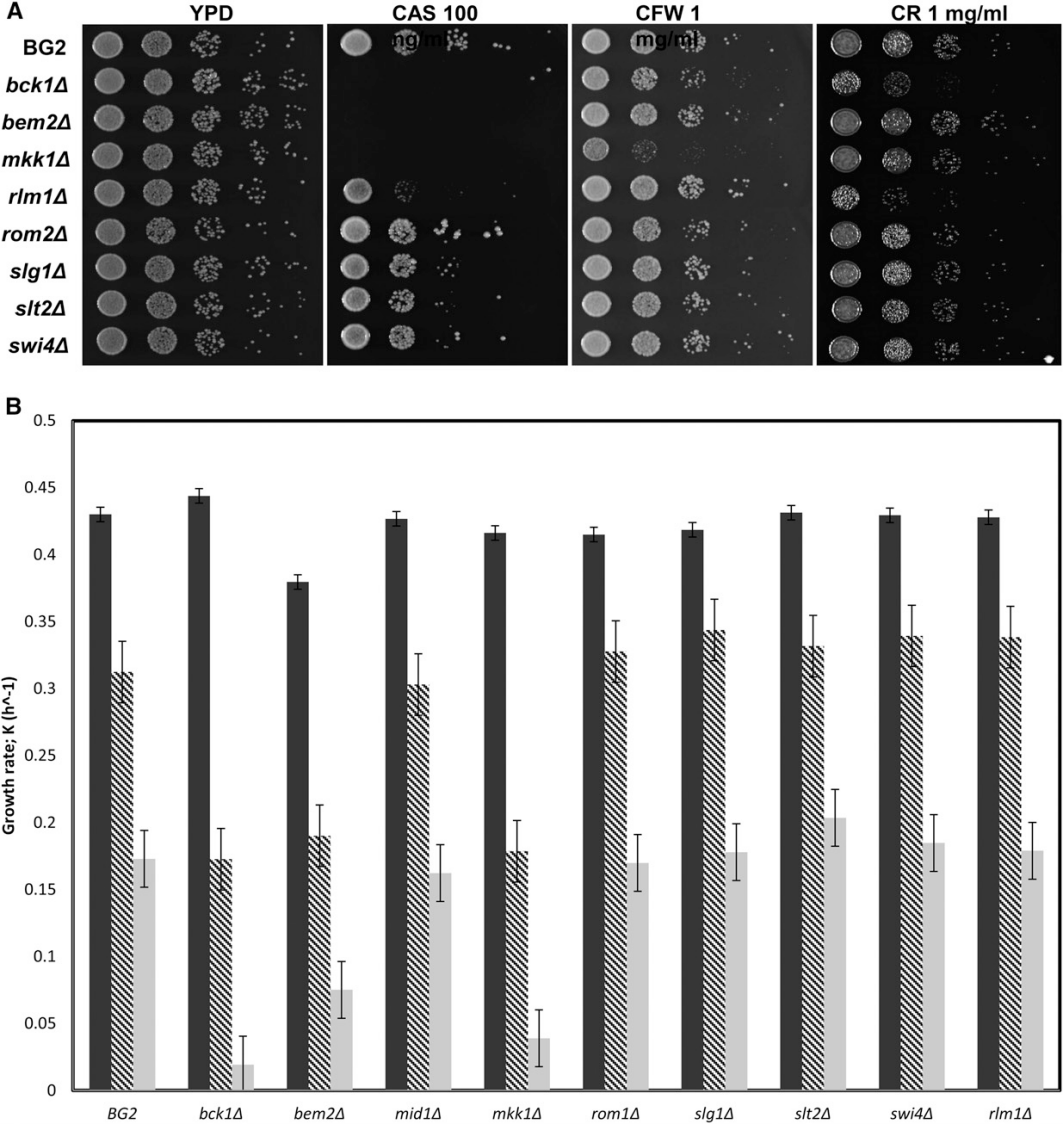

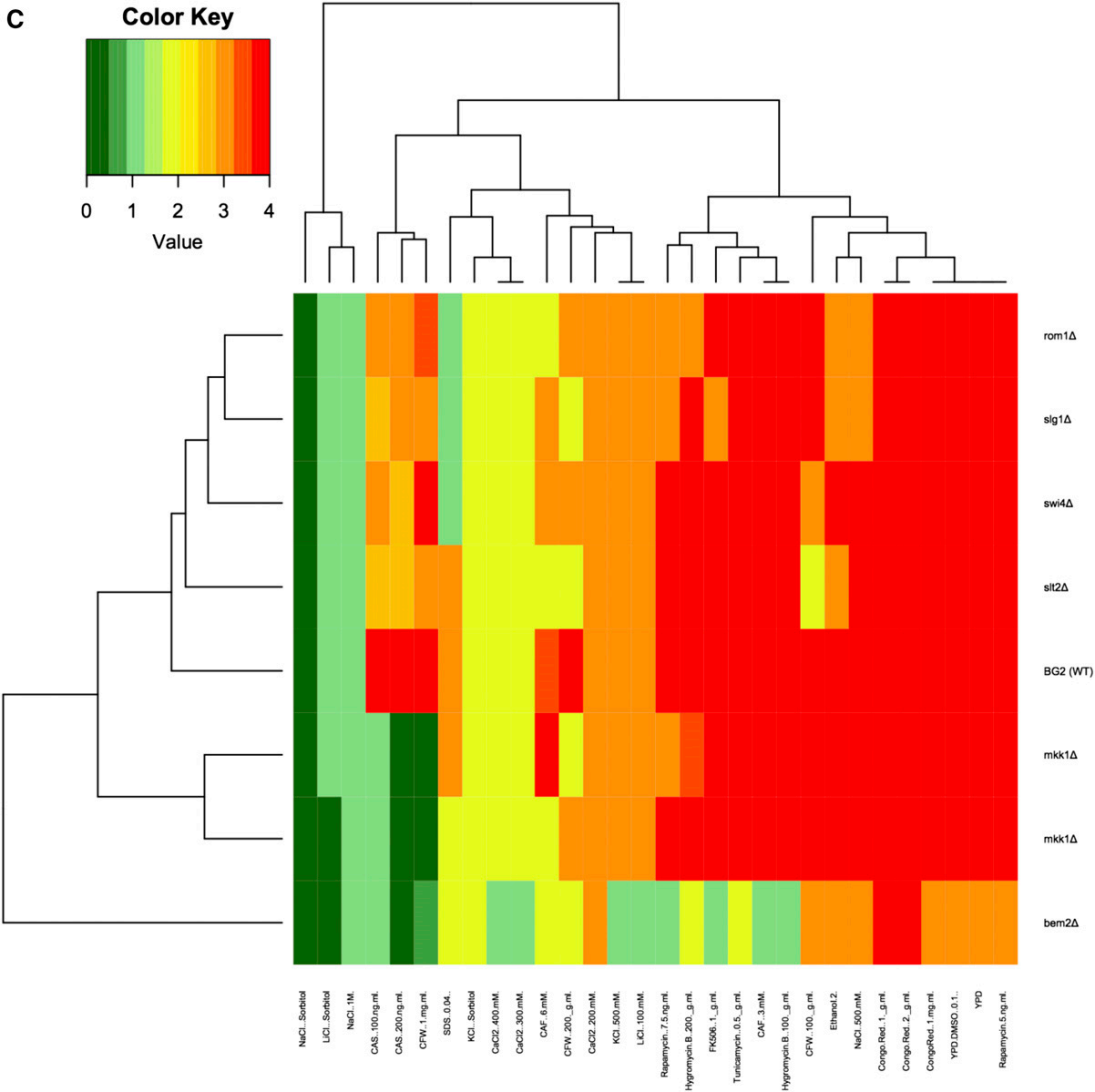
The CWI knockout mutants fall into two distinct groups based on growth in caspofungin. (A) CWI knockouts have altered sensitivity to caspofungin on plates. Complete knockout strains for CWI pathway genes, *bck1*Δ, *bem2*Δ, *mkk1*Δ, *rlm1*Δ, *rom2*Δ, *slg1*Δ, *slt2*Δ, and *swi6*Δ were spotted as 10-fold dilutions on YPD plates containing 100 ng/mL caspofungin (CAS), 1 mg/ml calcofluor white (CFW), 1 mg/ml Congo red (CR). The parental strain BG2 served as a comparison for growth. (B) Knockout strains have altered sensitivity to caspofungin in liquid medium. Strains with knockout of genes in the CWI pathway were grown with and without caspofungin as described in the legend to Figure 2C. (C) Phenotypes of the CWI pathway knockout mutants The nine knockout mutants constructed were examined on a variety of different media similar to those used to examine the phenotypes of the 64 Tn7 disruption mutants. These included: YPD, with or without 0.1% DMSO or 2% ethanol, 500 mM KCl, with and without 1 M sorbitol, 500 mM NaCl, with and without 1 M sorbitol, 1 M NaCl, 100 mM LiCl, with and without 1 M sorbitol, CaCl_2_ (200, 300, and 400 mM), calcofluor white (100 and 200 μg/ml, 1 mg/ml), caffeine (3 and 6 mM), Congo red (1 and 2 μg/ml, 1 mg/ml), rapamycin (5, 7.5, and 10 ng/ml, delivered in 0.1% DMSO), tunicamycin (0.5 μg/ml), FK506 (1 μg/ml), caspofungin (100 and 200 ng/ml), SDS (0.04%), and hygromycin B (100 and 200 μg/ml). The *bem2*Δ mutant was very sensitive to many of these conditions. Raw data are in Table S4.

We first tested each of the CWI knockout mutants on solid medium containing caspofungin, as well as in the presence of Calcofluor white and Congo red (Figure 3A). The mutants showed a range of sensitivities but fell more or less into two groups based on the caspofungin phenotype, with the *Cg bck1*Δ (CAGL0L03520g), *Cg bem2*Δ (CAGL0I06512g), and *Cg mkk1*Δ (CAGL0J03828g) mutants showing increased sensitivity relative to the *Cg rlm1*Δ (CAGL0H05621g), *Cg rom2*Δ (CAGL0G04873g), *Cg slg1*Δ (CAGL0F01507g), *Cg slt2*Δ (CAGL0J00539g), and *Cg swi4*Δ (CAGL0A04565g) mutant strains. The *Cg bck1*Δ (CAGL0L03520g), *Cg bem2*Δ (CAGL0I06512g), and *Cg mkk1*Δ (CAGL0J03828g) mutants were also slightly more sensitive to Calcofluor white than the others, while the *Cg bck1*Δ (CAGL0L03520g), and *Cg rlm1*Δ (CAGL0H05621g), mutant strains were sensitive to Congo red. Under liquid culture conditions (Figure 3B), the *Cg bck1*Δ (CAGL0L03520g), *Cg bem2*Δ (CAGL0I06512g), and *Cg mkk1*Δ (CAGL0J03828g) mutants were quite sensitive to caspofungin, while the other mutants appeared to have growth characteristics similar to wild type. In general however, the knockout mutant strains exhibited milder phenotypes than the Tn7 insertion mutants, suggesting that there may be *cis* effects stemming from the transposon into neighboring genomic DNA.

We examined the eight CWI knockout mutants under a variety of other conditions, similar to the tests used for the Tn7 disruption mutants isolated from the screen (Figure 3C summarizes these experiments; the complete data set is found in Table S4). As on plates and in liquid culture in the presence of caspofungin, the mutants fell into the same two groups based on these experiments. The *Cg rom2*Δ (CAGL0G04873g), *Cg rlm1*Δ (CAGL0H05621g), *Cg slg1*Δ (CAGL0F01507g), *Cg slt2*Δ (CAGL0J00539g), and *Cg swi4*Δ (CAGL0A04565g) mutants were mildly sensitive to many of the treatments, while the *Cg bem2*Δ (CAGL0I06512g), *Cg bck1*Δ (CAGL0L03520g), and *Cg mkk1*Δ (CAGL0J03828g) mutants were more sensitive. *The Cg bem2*Δ (CAGL0I06512g) mutant in particular was very sensitive to most treatments, including caspofungin, a variety of salts, and caffeine.

**Figure 4:**
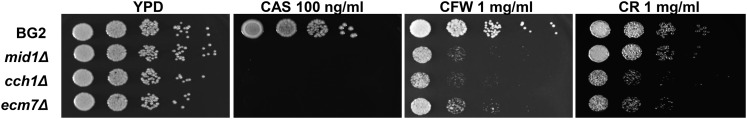
HACS complex knockouts display sensitivity to caspofungin, calcofluor white, and Congo red. Strains containing complete deletions of the HACS complex genes, *mid1*Δ, *cch1*Δ, and *ecm7*Δ were spotted as 10-fold dilutions on YPD plates containing 100 ng/mL caspofungin (CAS), 1 mg/ml calcofluor white (CFW), 1 mg/ml Congo red (CR). The parental strain BG2 served as a comparison for growth.

We also characterized strains with knockouts of presumptive HACS complex genes, *Cg cch1*Δ (CAGL0B02211g), *Cg ecm7*Δ (CAGL0M00748g), and *Cg mid1*Δ (CAGL0M03597g) (Figure 4). As with the Tn7 disruption mutants, the knockout strains were sensitive to caspofungin. In addition, the three mutant strains showed differential sensitivities to Calcofluor white [*Cg cch1*Δ (CAGL0B02211g) ανδ *Cg mid1*Δ (CAGL0M03597g) were slightly more sensitive than *Cg ecm7*Δ [CAGL0M00748g)] and Congo red [*Cg cch1*Δ (CAGL0B02211g) and *Cg ecm7*Δ (CAGL0M00748g) were more sensitive than *Cg mid1*Δ (CAGL0M03597g)].

To confirm that the gene we deleted was responsible for the phenotype, as proof-of-principle we cloned *Cg MID1* (CAGL0M03597g) into a low-copy *S. cerevisiae* vector, since such vectors function in *C. glabrata* (Zhou *et al.* 1994). The plasmid was transformed into the appropriate *Cg mid1*Δ (CAGL0M03597g) strain and phenotypes were examined. As shown in Figure S2, the cloned *MID1* (CAGL0M03597g) gene complemented the phenotype, thus assuring the phenotypes observed were due to the deletion of the gene of interest.

### Enzymatic cell wall digestion occurs more rapidly in CWI mutants

**Figure 5:**
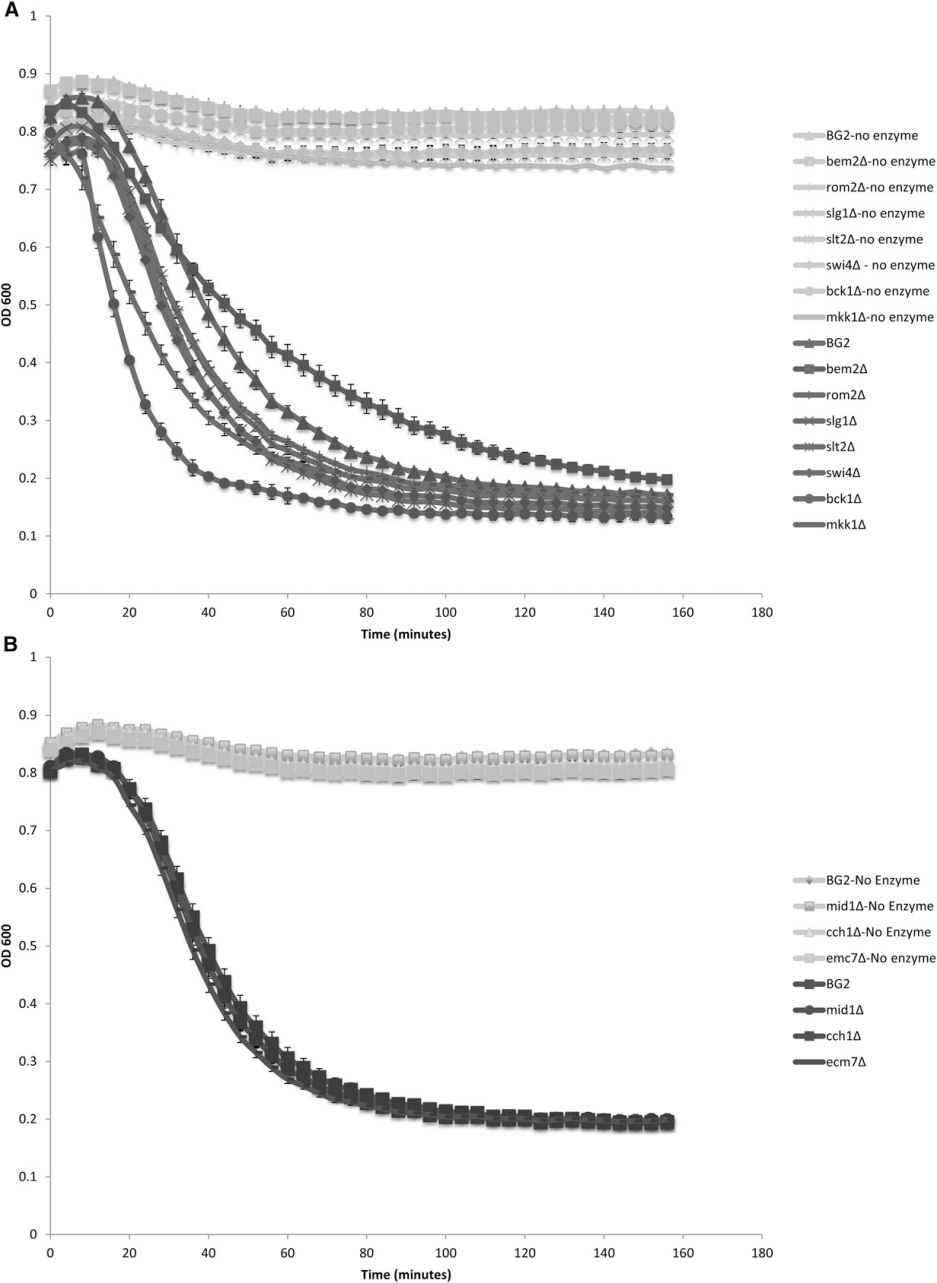
Cell wall mutants display sensitivity to zymolyase. Deletion mutants were tested for their sensitivity to cell wall digestion by treatment with zymolyase as described in *Materials and Methods*. (A) CWI pathway deletion mutants. (B) HACS deletion mutants

The cell wall normally protects the cell from osmotic pressure, but defects in, or damage to, the cell wall can lead to lysis. Cell wall integrity can be challenged by zymolyase, a cocktail of 1,3-β-glucanase and protease activities used to degrade yeast cell walls (Ovalle *et al.* 1998, 1999). Enzymatic digestion of cell walls in the absence of an osmotic stabilizer such as sorbitol results in lysis due to increased internal turgor pressure (Ovalle *et al.* 1998, 1999). We compared the sensitivity of the deletion mutant strains to zymolyase (Figure 5). Strains with deletions of CWI orthologs had a greater sensitivity to zymolyase, particularly the *bck1*Δ (CAGL0L03520g) strain (Figure 5A). Interestingly, the *bem2*Δ (CAGL0I06512g) strain displayed lower sensitivity (Figure 5A), despite the fact that the *bem2*Δ (CAGL0I06512g) strain was very sensitive to caspofungin, and other cell-well disrupting agents (Figure 3C). In contrast, strains containing deletions of HACS complex orthologs demonstrated the same level of sensitivity as the wild-type BG2 strain (Figure 5B), suggesting that the changes that result in caspofungin sensitivity in these strains is not the result of changes in glucan content.

## Discussion

The work described here represents a global approach to identifying genes important for cellular responses to caspofungin in *C. glabrata*. Our results, taken all together, demonstrate that many different genes impact cell wall structure and function in *C. glabrata*, including members of the CWI pathway and the HACS complex.

Similar screens have been performed with caspofungin in *S. cerevisiae* (Lesage *et al.* 2004; Markovich *et al.* 2004), using the yeast deletion collection (Winzeler *et al.* 1999), and with micafungin, another member of the echinocandin family in *Schizosaccharomyces pombe* (Zhou *et al.* 2013), using the *S. pombe* deletion collection (Kim *et al.* 2010). In addition, microarray analysis identified genes in *S. cerevisiae* that are up or downregulated in response to caspofungin (Reinoso-Martin *et al.* 2003). Most recently, a large collection of deletion strains (representing 600+ genes) was screened for caspofungin sensitivity in *C. glabrata* (Schwarzmuller *et al.* 2014). However, in comparing the lists of genes identified in each instance, numbers of different genes were identified, even between the two screens performed in *S. cerevisiae* (Lesage *et al.* 2004; Markovich *et al.* 2004). Nevertheless, in instances where the same gene was found in different screens, mutant strains were observed to have the same general phenotype. For example, mutation of *MNN10*, which encodes a subunit of the Golgi apparatus mannosyltransferase complex, was found to result in sensitivity to caspofungin in *S. cerevisiae* (Lesage *et al.* 2004; Markovich *et al.* 2004), as well as in our study, and the deletion collection screen in *C. glabrata* (Schwarzmuller *et al.* 2014). The sole exception to this trend was mutation of CRZ1, a transcription factor that is regulated by calcineurin. We found that disruption with Tn7 results in sensitivity to caspofungin in *C. glabrata*, a result supported by the work of Miyazaki *et al.* (2010b) in *S. cerevisiae*, whereas Lesage *et al.* (2004) found that deletion of *CRZ1* in *S. cerevisiae* results in resistance.

Despite the fact that incompletely overlapping sets of genes were identified in the various screens, in each of these reports, genes controlling the CWI pathway were identified. In our case, these genes included the *C. glabrata* orthologs of *BEM2* (CAGL0I06512g), *MKK1* (CAGL0J03828g), *SLT2* (CAGL0J03828g), and *SWI4* (CAGL0A04565g). Similarly, in the screen of the 600+ members of the *C. glabrata* deletion collection, the *C. glabrata* orthologs of *MKK1* (CAGL0J03828g), *BCK1* (CAGL0L03520g), *SLT2* (CAGL0J00539g), and *SLG1/WSC1* (CAGL0F01507g) were identified (Schwarzmuller *et al.* 2014). Further, we here demonstrated that strains with deletions of *BCK1* (CAGL0L03520g), *RLM1* (CAGL0H05621g), *ROM2* (CAGL0G04873g), or *SLG1* (CAGL0F01507g) were also sensitive to caspofungin, although to varying degrees.

Other groups previously investigated individual genes involved in the CWI pathway in *C. glabrata*. First, it was demonstrated that deletion of *Cg SLT2* (CAGL0J00539g), encoding a serine/threonine kinase, results in increased sensitivity to cell wall stress, including the presence of caspofungin (Miyazaki *et al.* 2010a), micafungin (Miyazaki *et al.* 2010a; Nagayoshi *et al.* 2014), Calcofluor white (Edlind *et al.* 2002), and fluconazole (Borah *et al.* 2011), while overexpression of *Cg SLT2* (CAGL0J00539g) resulted in tolerance—findings that correlated with decreased or increased ability to survive in a mouse host, respectively (Edlind *et al.* 2002; Cota *et al.* 2008). In addition, the overexpression of *Cg SLT2* (CAGL0J00539g) leads to increases in chitin content, correlating with poor killing by caspofungin (Cota *et al.* 2008). Similarly, deletion of *Cg SWI4* (CAGL0A04565g), *Cg SWI6* (CAGL0B01144g), and *Cg RLM1* (CAGL0H05621g), transcription factors that are activated as a result of the CWI pathway, results in sensitivity to micafungin, another member of the echinocandin family, and deletion of *Cg SWI4* (CAGL0A04565g) results in sensitivity to caspofungin (Nagayoshi *et al.* 2014). Thus it seems clear that mutations in CWI pathway genes enhance the efficacy of caspofungin. Such results suggest that chemical modulators of the CWI pathway may increase the efficacy of caspofungin as well. Indeed, it has been reported that *S. cerevisiae* strains with deletions of CWI pathway genes *Cg BCK1* (CAGL0L03520g) and *Cg SLT2* (CAGL0J00539g) are sensitive to the anti-malarial drug chloroquine, and that chloroquine and caspofungin show synergy in *S. cerevisiae*, *C. albicans*, and *A. fumigatus*, as well as *C. glabrata* (Islahudin *et al.* 2013).

Regulation of chitin content also appears to be a means of regulating responses to echinocandins. Increased abundance of chitin correlates with echinocandin resistance in *C*. *albicans* (Walker *et al.* 2013). In contrast, deletion of *Cg CHS3B* (CAGL0I04840g)—a paralog of chitin synthase 3—results in sensitivity to several different echinocandins, including micafungin and nikkomycin Z, as well as caspofungin (Ueno *et al.* 2011).

Inhibition of Ca^2+^ influx via the HACS complex may also increase the efficacy of caspofungin. As shown in Figure 2, we obtained Tn7 disruption mutants in each of the three HACS complex members, *Cg CCH1* (CAGL0B02211g), *Cg ECM7* (CAGL0M00748g), and *Cg MID1* (CAGL0M03597g)—results we verified by creating deletion mutants (Figure 4). Others have shown that *ECM7* is important in *C. albicans* (orf19.5643) for responses to oxidative stress and Ca^2+^ homeostasis (Ding *et al.* 2013). Previous work in *S. cerevisiae* demonstrated that *FKS2* is regulated not only via the CWI pathway, but also as a result of Ca^2+^ flux, mediated by calmodulin, calcineurin, and Crz1 (Zhao *et al.* 1998; Levin 2011). Cross-talk between this pathway and the CWI pathway might occur at some level; for example, it is tempting to speculate that protein kinase C, Pkc1 might also bind and be activated by Ca^2+^, since it has a C2-like domain (Rizo and Sudhof 1998), but purified *S. cerevisiae* Pkc1 tested *in vitro* is unaffected by Ca^2+^ (Antonsson *et al.* 1994; Watanabe *et al.* 1994).

There are two other proteins, Kch1 and Kch2 , which act as low-affinity transporters of K^+^ (Stefan *et al.* 2013), that are known to be important for the activity of the HACS complex in *S. cerevisiae*. The activity of Kch1 and Kch2 may set up a voltage differential across the plasma membrane, thus activating the HACS complex. Kch1 is conserved in *C. albicans* (orf19.6563) (Stefan and Cunningham 2013), and in *C. glabrata* (CAGL0D06050g) (Inglis *et al*. 2012). However, we did not obtain the *C. glabrata* ortholog in our screen. Similarly, strains with deletions in glycerol channels, *Cg FPS1* (CAGL0C03267g) or *Cg FPS2* (CAGL0E03894g) (Beese-Sims *et al.* 2012) are sensitive to caspofungin, yet we did not isolate these in our screen. Thus, although the Tn7 insertion collection is extensive, not every nonessential gene in the genome may be represented. This likely explains why we did not isolate all of the nonessential CWI pathway genes in the screen or any of the chitin synthase genes, for example. Nevertheless, we have identified a number of different pathways and complexes that, when disrupted, lead to altered sensitivity to caspofungin. We plan to further investigate the hits obtained from this study, particularly the genes involved in mannan synthesis, including *ALG6* (CAGL0E02629g), *DFG10* (CAGL0L00693g), *MNN5* (CAGL0M02871g), *MNN10* (CAGL0K11231g), *PMT2* (CAGL0J08734g), and *VAN1* (CAGL0B02321g) (Table 1 and Table 2).

We also identified a number of genes that, when disrupted, result in increased tolerance to caspofungin compared to wild type. None of these genes are currently associated with clinical resistance; rather resistance in the clinic is associated primarily with mutations in *FKS1* and *FKS2* (Arendrup *et al.* 2012; Duran-Valle *et al.* 2012; Singh-Babak *et al.* 2012; Alexander *et al.* 2013; Borghi *et al.* 2014; Pham *et al.* 2014). Nevertheless, it will be important to know what other mutations can cause resistance to echinocandins, given that these drugs are among the few available to treat many *C. glabrata* infections.

## Supplementary Material

Supplemental Material
