# Supplementary material for: Identification of Genes in Candida glabrata Conferring Altered Responses to Caspofungin, a Cell Wall Synthesis Inhibitor
Source: G3 (Bethesda). 2016 Jul 21;6(9):2893–907. doi: 10.1534/g3.116.032490 (PMC5015946; doi:10.1534/g3.116.032490)
Supplement: Supplemental Material [file supp_6_9_2893__index.html]

Identification of Genes in Candida glabrata Conferring Altered Responses to Caspofungin, a Cell Wall Synthesis Inhibitor — Supplemental Material 

# Identification of Genes in *Candida glabrata* Conferring Altered Responses to Caspofungin, a Cell Wall Synthesis Inhibitor

## Supplemental Material for Rosenwald *et al.*, 2016

**Files in this Data Supplement:**

- Figure S1 - Example screen results. (.pdf, 175 KB)
- Figure S2 - Complementation of the *Cg mid1Δ* deletion. (.pdf, 191 KB)
- Table S1 - GO terms associated with genes in the selected set. (.pdf, 25 KB)
- Table S2 - Genes knocked out in the BG14 background. (.pdf, 195 KB)
- Table S3 - Raw data for figure 2A. (.xlsx, 17 KB)
- Table S4 - Raw data for figure 3C. (.xlsx, 46 KB)
