# Supplementary material for: Identification of Genes in Candida glabrata Conferring Altered Responses to Caspofungin, a Cell Wall Synthesis Inhibitor
Source: G3 (Bethesda). 2016 Jul 21;6(9):2893–907. doi: 10.1534/g3.116.032490 (PMC5015946; doi:10.1534/g3.116.032490)

Figure S1. Example screen results

Replica prints of plate T27, one of the 270 96-well dishes containing the *C. glabrata* transposon collection. “YPD” (leftmost panel) is regular growth medium without drug, “100” contains 100 ng/ml (middle panel) and “200” contains 200 ng/ml caspofungin (rightmost panel). As shown by the arrows, this particular plate has several clones that grow poorly in 100 ng/ml caspofungin; and several others that grow well in 200 ng/ml.

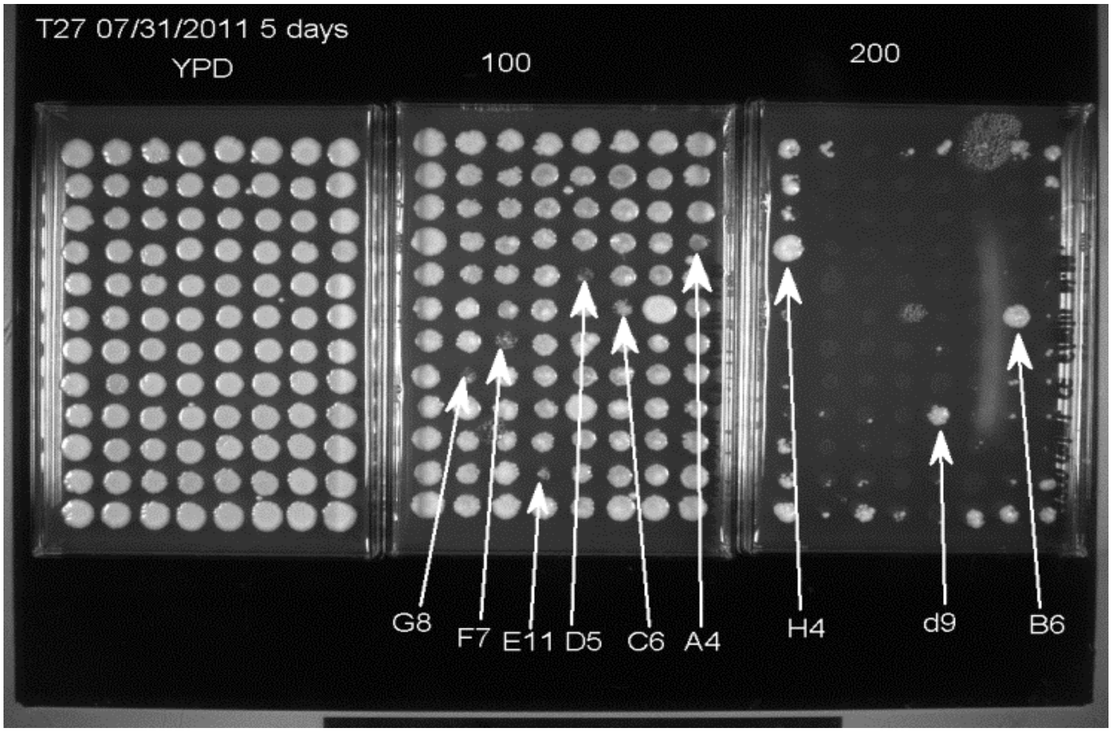

Supplement: Supplemental Material [file supp_g3.116.032490_FigureS1.pdf]
