# Supplementary material for: Identification of Genes in Candida glabrata Conferring Altered Responses to Caspofungin, a Cell Wall Synthesis Inhibitor
Source: G3 (Bethesda). 2016 Jul 21;6(9):2893–907. doi: 10.1534/g3.116.032490 (PMC5015946; doi:10.1534/g3.116.032490)

Figure S2. Complementation of the *Cg mid1Δ* deletion

As described in Methods, *Cg MID1* was cloned into a vector containing the Nat resistance genes. The *Cg mid1Δ* strain was transformed with empty vector or vector containing wild type *Cg MID1*. *MID1* complements the caspofungin sensitivity phenotype.

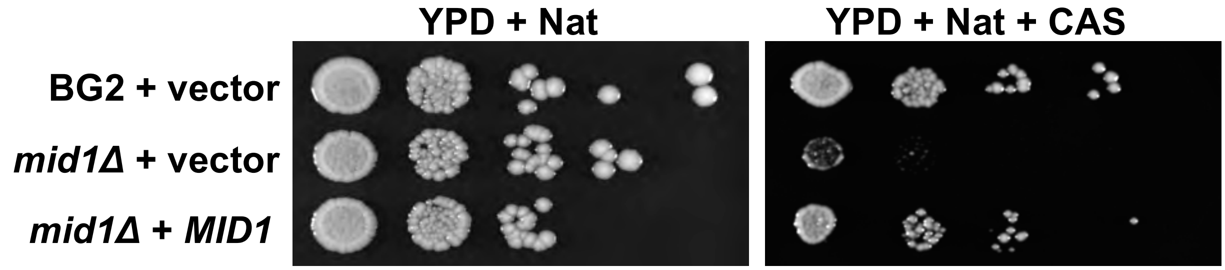

Supplement: Supplemental Material [file supp_g3.116.032490_FigureS2.pdf]
