# Supplementary material for: Identification of Genes in Candida glabrata Conferring Altered Responses to Caspofungin, a Cell Wall Synthesis Inhibitor
Source: G3 (Bethesda). 2016 Jul 21;6(9):2893–907. doi: 10.1534/g3.116.032490 (PMC5015946; doi:10.1534/g3.116.032490)
Supplement: Supplemental Material [file supp_g3.116.032490_TableS1.pdf]

Table S1. GO terms associated with genes in the selected set

As described in the legend to Figure 1, 64 *C. glabrata* orthologs of *S. cerevisiae* genes identified in the caspofungin screen were submitted to BinGO analysis (**A**) and to FunSpec analysis (**B**; the Biological Process results are shown, run without and with Bonferroni correction). Sixty-three gene names are associated with the GO terms as listed here. x = number of genes from the test set matching that GO term, while n = total number of genes in the genome with that term. p-value is the statistical significance associated with the number of genes in the test set relative to the total number of genes with a given GO term. *TAD1* is not associated with any GO terms and is therefore not represented in the table. Genes that when disrupted cause caspofungin sensitivity are in black; genes that when disrupted cause caspofungin tolerance are in red.

**A. BinGO Analysis** **GO Term Description**	**GO-ID**	**X**	**N**	**p-value**	**Genes in test set**
cell wall organization	71555	11	254	4.04E-05	*BEM2 CNA1 CRR1 ECM7 GAS1 LRG1 MNN10 PST1 SLT2 SWI4 VAN1*
signal transduction	7165	10	259	2.44E-04	*BEM2 BFA1 CRZ1 GPR1 KSS1 LRG1 MKK1 MSG5 SLT2 SRO7*
vesicle-mediated transport	16192	12	370	2.82E-04	*DNF1 FTH1 GEA2 GGA2 INP53 PEP1 PMR1 SCD5 SHR3 SLT2 SRO7 STV1*
Membrane	16020	32	1852	3.70E-04	*ALG6 BEM2 BPT1 CCH1 DFG10 DNF1 ECM7 FTH1 GAS1 GEA2 GGA2 GPR1 GTR2 ITR2 MID1 MNN10 MTC4 ORM1 PAT1 PDR16 PEP1 PMR1 PMT2 PST1 SCD5 SHR3 SLT2 SPO7 SRO7 STV1 VAN1 ZRT3*
Golgi apparatus	5794	9	242	6.74E-04	*GEA2 GGA2 MNN10 MNN5 PEP1 PMR1 STV1 VAN1 YPR089W*
transporter activity	5215	11	392	1.73E-03	*BPT1 CCH1 DNF1 FTH1 ITR2 MID1 PAT1 PDR16 PMR1 STV1 ZRT3*
Transport	6810	22	1221	3.18E-03	*BPT1 CCH1 DNF1 FTH1 GEA2 GGA2 GTR2 INP53 ITR2 LRG1 MID1 PAT1 PEP1 PMR1 PDR16 SCD5 SHR3 SLT2 SRO7 STV1 YDJ1 ZRT3*

protein modification process	6464	14	639	4.09E-03	*ALG6 ECM7 KSS1 MKK1 MNN10 MNN5 MSG5 PMT2 RAD5 RTT109 SHR3 SIN3 SLT2 VAN1*
signal transducer activity	4871	4	70	5.36E-03	*GPR1 KSS1 PEP1 SLT2*
pseudohyphal growth	7124	4	73	6.22E-03	*DFG10 FKH2 GPR1 KSS1*
response to chemical stimulus	42221	10	399	6.40E-03	*CNA1 GPR1 KSS1 MKK1 MSG5 ORM1 PDR16 SLT2 YKL075C WAR1*
**B. FunSpec Analysis** **GO Term Description** (without Bonferroni correction)	**GO-ID**	**X**	**N**	**p-value**	**Genes in test set**
Calcium ion transport	6816	4	7	2.75E-07	*CCH1 ECM7 MID1 PMR1*
Calcium ion transmembrane transport	70588	2	4	5.48E-04	*CCH1 MID1*
Ion transport	6811	6	107	5.50E-04	*CCH1 MID1 PMR1 STV1 ZRT3*
Signal transduction	7165	5	73	6.60E-04	*BEM2 LRG1 MKK1 SLT2*
**GO Term Description** (with Bonferroni correction)	6816	4	7	1.59E-07	*CCH1 ECM7 MID1 PMR1*
