# Supplementary material for: Identification of Genes in Candida glabrata Conferring Altered Responses to Caspofungin, a Cell Wall Synthesis Inhibitor
Source: G3 (Bethesda). 2016 Jul 21;6(9):2893–907. doi: 10.1534/g3.116.032490 (PMC5015946; doi:10.1534/g3.116.032490)
Supplement: Supplemental Material [file supp_g3.116.032490_TableS2.pdf]

Table S2. Genes knocked out in the BG14 background

C gla Locus Tag	*S cer* Homolog	Gene Name	Description of gene product
			PKC/Cell Wall Integrity Pathway
CAGL0L03520g	YJL095W	*BCK1*	Kinase in PKC-MAPK pathway
CAGL0I06512g	YER155c	*BEM2*	RhoGAP involved in the control of cytoskeleton organization
CAGL0J03828g	YPL140C	*MKK1*	MAPKK involved in control of cell integrity
CAGL0H05621g	YPL089C	*RLM1*	MADS-box transcription factor; component of the CWI pathway
CAGL0G04873g	YLR371W	*ROM2*	GEF for Rho1/2
CAGL0J00539g	YHR030c	*SLT2*	Serine/threonine MAP kinase; regulates cell wall integrity
CAGL0F01507g	YOR008C	*SLG1*	Sensor-transducer of the PKC-MAPK pathway
CAGL0A04565g	YER111c	*SWI4*	DNA binding component of the SBF complex
			High Affinity Ca^{2+} Influx (HACS)
CAGL0B02211g	YGR217W	*CCH1*	Ca^{2+}-permeable cation channel required for Ca^{2+} influx
CAGL0M00748g	YLR443W	*ECM7*	Ca^{2+}-permeable cation channel required for Ca^{2+} influx
CAGL0M03597g	YNL291C	*MID1*	Ca^{2+}-permeable cation channel required for Ca^{2+} influx
